# Application of holography and augmented reality based technology to visualize the internal structure of the dental root – a proof of concept

**DOI:** 10.1186/s13005-022-00307-4

**Published:** 2022-04-05

**Authors:** Damian Dolega-Dolegowski, Klaudia Proniewska, Magdalena Dolega-Dolegowska, Agnieszka Pregowska, Justyna Hajto-Bryk, Mariusz Trojak, Jakub Chmiel, Piotr Walecki, Piotr S. Fudalej

**Affiliations:** 1 3D Medical IT, Kraków, Poland; 2 Jagiellonian University Medical College, Kraków, Poland; 3 Centrum Medyczne Liderdent, Kraków, Poland; 4 Institute of Fundamental Technological Research, Polish Academy of Sciences, Warsaw, Poland; 5 Jagiellonian University, Kraków, Poland; 6 Department of Cardiac and Vascular Diseases, Jagiellonian University Medical College, Krakow, Poland; 7 Institute of Dentistry and Oral Sciences, Faculty of Medicine and Dentistry, Palacký University Olomouc, Olomouc, Czech Republic; 8 Institute of Dentistry, Jagiellonian University Medical College, Kraków, Poland; 9 Department of Orthodontics and Dentofacial Orthopedics, University of Bern, Bern, Switzerland

**Keywords:** Mixed reality, Augmented reality, Holography, Tooth, Dental root, Root canal, Visualization

## Abstract

**Background:**

The Augmented Reality (AR) blends digital information with the real world. Thanks to cameras, sensors, and displays it can supplement the physical world with holographic images. Nowadays, the applications of AR range from navigated surgery to vehicle navigation.

**Development:**

The purpose of this feasibility study was to develop an AR holographic system implementing Vertucci’s classification of dental root morphology to facilitate the study of tooth anatomy. It was tailored to run on the AR HoloLens 2 (Microsoft) glasses. The 3D tooth models were created in Autodesk Maya and exported to Unity software. The holograms of dental roots can be projected in a natural setting of the dental office. The application allowed to display 3D objects in such a way that they could be rotated, zoomed in/out, and penetrated. The advantage of the proposed approach was that students could learn a 3D internal anatomy of the teeth without environmental visual restrictions.

**Conclusions:**

It is feasible to visualize internal dental root anatomy with AR holographic system. AR holograms seem to be attractive adjunct for learning of root anatomy.

## Introduction

Dental students have to learn dental anatomy for the successful diagnosis and treatment of different pathological processes. Currently, students have a wide range of books, videos, lectures, and seminars from which they can study. However, recent technological advancements offer new possibilities for improvement of the effectiveness of the learning process [1, 2].

In 2016, Microsoft released a product called “HoloLens” (www.microsoft.com/en-us/hololens). It is a device resembling goggles with a built-in battery, microcomputer, cameras, microphone, speakers, and holographic projectors. Installed depth cameras analyze the entire environment around the user (e.g. position and shape of tables, chairs, walls, etc.) and the microcomputer decides about a way of displaying a hologram so that it is compatible with the environment. This enhancement of the real world by computer-generated information is called augmented reality (AR). Virtual reality (VR), as opposed to AR, replaces the surrounding reality with the digital world so that users’ perception of reality is entirely based on virtual information. With continuous improvement of the performance of head-mounted displays, the potential of VR and AR is increasing - they are more and more often used in medicine [3, 4], dentistry [5–8], and education in health sciences [9–12].

In endodontics, knowledge of the internal anatomy of teeth is instrumental. Students and clinicians must have a well-developed spatial vision to imagine the course of root canals when they start endodontic treatment. Attempts to utilize VR [13] or HoloHuman application [14] for teaching head anatomy demonstrated potential of holographic technologies as an adjunct tool in this field. Moreover, Song et al. [15] introduced the general idea of endodontic treatment assisted by AR-guided technologies. Thus, the purpose of this paper was to describe the development of a Microsoft HoloLens 2-based application enabling 3D display of the internal anatomy of dental roots for facilitation of learning process.

### Development process

#### Holography

First reports about early holographic technology date back to 19th century [16]. Later, hologram was defined as an interference pattern between a coherent reference beam and the wave, which has been scattered by the real object [17]. Recent rapid and versatile development of holographic technology [18] holds great promise for medical education because it enables to visualize complex structures or procedures taught during classes.

#### Augmented reality

AR is an enhancement of the real world by computer-generated information. Using AR glasses, the user sees the environment he/she is in and the glasses display holograms harmonizing with the setting. A considerable benefit of AR in comparison to VR is that the user never loses orientation in the environment (frequent for someone using VR devices).

**Fig. 1:**
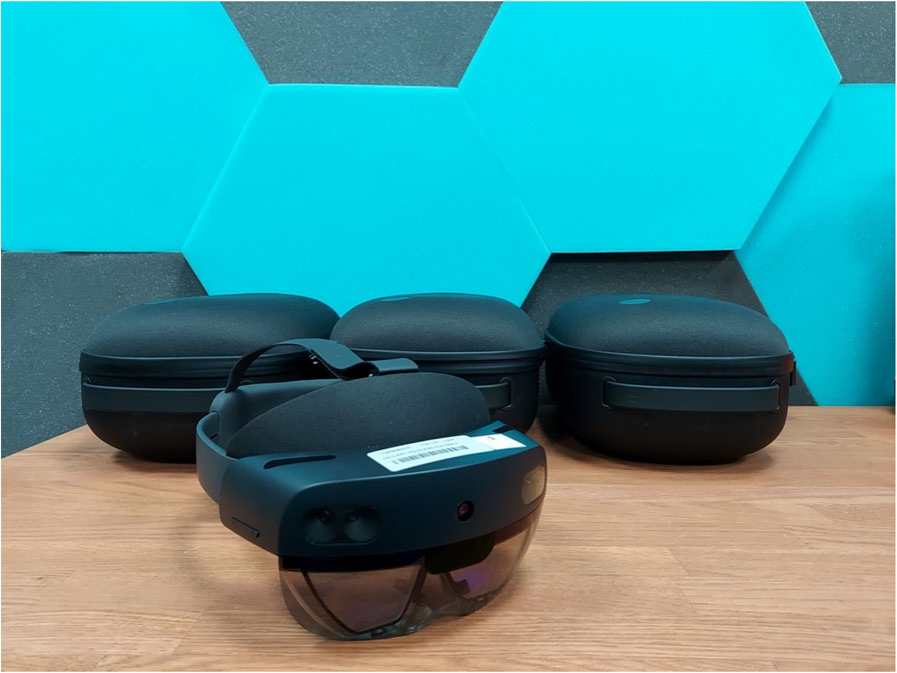
HoloLens 2 glasses

The HoloLens 2 (*Microsoft, Redmond, USA*) (Fig. 1) is a commercially available system of AR. It is an independent device and does not require a separate operation space and manual controllers. It is fully integrated with Microsoft Enterprise systems, which resembles other Microsoft operating systems (e.g. Windows 10). The disadvantage of HoloLens 2 is that the commercial license is currently rather expensive [19]. Some educational trainings were already developed [20–22].

### Development of the application

We planned to implement all holographic features offered by the HoloLens 2 device: (a) ability to display images truly 3-dimensionally – they are projected as 3D objects in front of the user so that he/she can “feel” them. It is a significant progress in comparison to today’s standard according to which 3D models are displayed only in 2D views. PC’s laptops, tablets, and books offer the option to view the 3D model but only in a two-dimensional perspective. One can rotate or view models from different angles, but these are only “3D-like” views because they are shown on a flat screen/page; (b) display of 3D models in such a way that they look like they are displayed in a specific area of the space. For example, one can force a device to display a model above the table and then walk around the table but with the model always “hanging” in the same place above the table. This feature of HoloLens 2 allows watching the 3D models from any perspective, angle, or place; (c) change of view perspective depending on the angle of observation; (d) automatic scanning of the environment to determine where the 3D model(s) would be placed in the space; (e) possibility that a user can look into the 3D object just by moving his/her head into it.

**Fig. 2:**
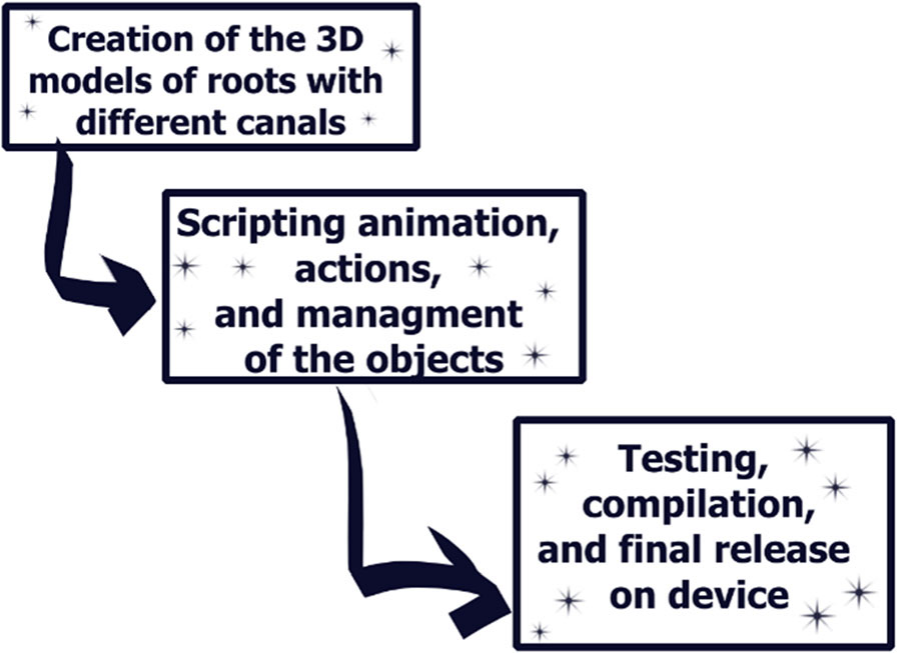
A scheme of the HoloLens-based application development

The development process included several steps (Fig. 2):

**Fig. 3:**
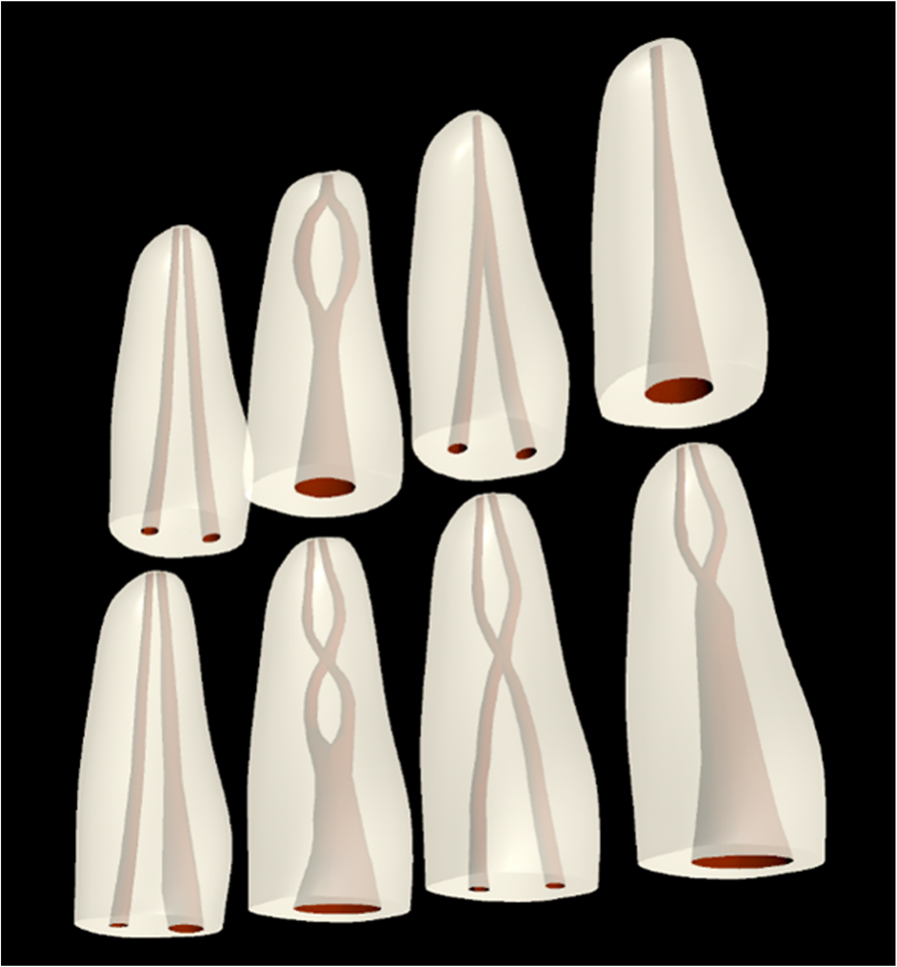
The root canal configurations from the pulp chamber to the root apex according to Vertucci [23] displayed with our application utilizing the HoloLens device

a) development of the 3D model of a root of the tooth (maxillary 1st. premolar) with a different pattern of course of the canal. The 3D model had to define connections, separations, or change in the direction of the root canal(s). It was prepared accordingly to Vertucci’s classification [23] - (Fig. 3). The entire work was done using Autodesk Maya – software used to create 3D models for games and animations. When the model was done, it was exported to Unity software - an application used to create video games and AR software for different devices and systems. A large benefit of this application is that it allows creating AR applications/games for the HoloLens 2 device. Thanks to it, the model created in Maya could be imported to the project;

b) creation of scripts. The scripts were needed to:

- place a model (root) in the exact position in the user-visible area (anchoring the model in the environment).
- animate action for “clicked”/selected object (root) in the way that it moved to the middle of the screen, increased its size, and started to rotate.
- animate action for returning the model (root) to its original place and to mark it as already used/selected.
- perform ‘main management’, i.e., the task of identifying which root was selected and determining where it should return;

c) testing the application in Unity emulator twitch export ready project with Microsoft Visual Studio. This development software is responsible for the final compilation process in the way that the application could be installed on the Microsoft HoloLens 2 device.

**Fig. 4:**
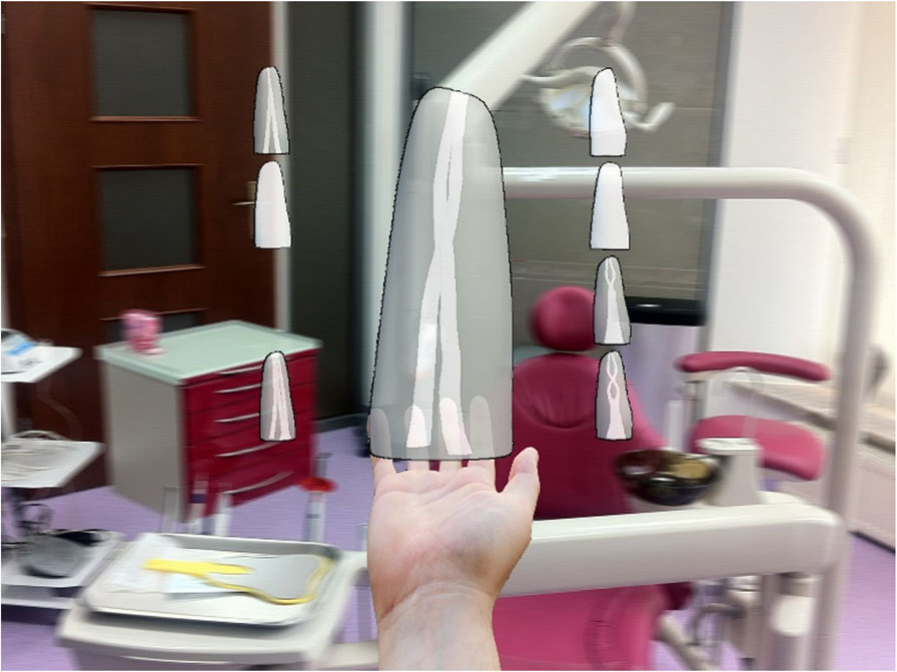
Experimental holograms projected in the operating room of the dental office

We created the 3D model of the tooth using the above-described steps. Then, the model was extended to include several types of root canal morphology according to [23]. Then, the 3D models were exported into the Unity application. Each model was filled with a script responsible for doing the programmed action. As a result, when the user starts the application, he/she can see 8 root models, each with different morphology and course of the canal. After pointing and triggering a click with the finger, the selected root model moves to the front of the user, changes its size by around 3 times, and starts to rotate slowly. In the background, the user can hear the lector gives a short definition of the selected model. The user has also an option to point to the model and to trigger a click to stop the rotation of the model. Another click would force the model to go back to its original size and to return to its original place in the row and column. This way the user can feel as if he/she took a root of the tooth from the shelf, watched, returned, and took possibly another one if need be (Fig. 4).

### Initial evaluation

Holograms of dental roots projected in a natural setting of the dental office (Fig. 4) and showed to dental school instructors and dental students provoked great interest. All participants of the presentation of the application were amazed by the holograms of the root models. Everyone agreed that holography and AR technologies could offer a significant opportunity to facilitate the learning process during dental studies.

The formal part of the initial evaluation of the application consisted of 2 components: (1) demonstration of the application and (2) answering a questionnaire (Table 1). During the demonstration, each evaluator was required to wear a HoloLens 2 headset and manipulate the displayed dental anatomy models themselves. During the demonstration, a member of the research team was always present to assist the raters, answer questions, and resolve technical issues if they arose. The demonstration was conducted once. The characteristics of the evaluation panel are shown in Table 2.

**Table 1:** The questionnaire given to the evaluators to complete along with the scores for each question

Questions	Answer	Students (*n* = 6)	Dentists (*n* = 6)
In your opinion the use of HoloLens 2 goggles was…			
	easy	6	5
	a little bit complicated	0	1
	difficult	0	0
The visualization of the root canal was…			
	clear and legible	2	6
	certain areas were blurry, but overall the quality was acceptable	4	0
	blurry and difficult to orientate	0	0
The visualization with HoloLens 2 goggles provides…			
	more information about the anatomy of the canal than visualization on a computer screen	4	6
	as much information about the canal anatomy as visualization on a computer screen	2	0
	less information about the canal anatomy than visualization on a computer screen	0	0
Do you think that visualization of the root canal anatomy…			
	can improve the effectiveness of endodontic treatment?	6	6
	will not improve the effectiveness of endodontic treatment?	0	0
	can reduce the effectiveness of endodontic treatment?	0	0
Do you think that the visualization of the canal anatomy shown to the PATIENT…			
	reduces his/her stress during endodontic treatment?	3	4
	does not affect the level of stress during endodontic treatment?	2	2
	increases his/her stress during endodontic treatment?	1	0
Your professional status			
	student	6	
	dentist	6	
Your gender			
	male	3	
	female	9	
	other	0	
Your age		28.2 years

**Table 2:** The demographic data of the evaluators

	Dental students (*n* = 6)	Dentists (*n* = 6)
Age in years (mean, SD, range)	20.7/2.0/18.0 - 24.0	35.7/4.0/29.0 - 42.0
Sex (M/F)	2 / 4	1 / 5

The questionnaire included 8 questions-5 of which were related to the prepared application, and the others described the status of the rater (student vs. physician, gender, and age). The questionnaire was available online at the demonstration site, and evaluators were asked to complete it immediately after the demonstration. The results of the assessment are shown in Table 1.

## Discussion and conclusions

In this paper, we present the application of holography and AR for visualizing the internal structure of the dental root. The use of this technology in medicine, especially dentistry, is a relatively new approach. The first commercial course using AR for teaching purposes was published in 2016 by Case Western Reserve University School of Medicine (Cleveland, OH, USA) in collaboration with Cleveland Clinic (Cleveland, Ohio) [24]. Their HoloAnatomy allowed the body and its structures, systems, and organs to be rotated and virtually dissected. Compared to traditional cadaver dissection, this is an effective and time-saving method for teaching anatomy [25]. Our team has also actively used the HoloAnatomy application in teaching medical and dental students. Another mixed reality (MR)-based application, HoloPatient, is a learning tool for nurses [26]. Recently, advances have also been made in the field of applying immersive technologies (VR, AR, and MR) to dentistry [27]. Jiang et al. [28] presented an AR-based application that allowed interactive display of the planned preparation curves of adjacent teeth. An interesting study on a three-dimensional AR system with integrated videography for displaying oral and maxillary regions was presented by Suenaga et al. [29]. It allowed stereoscopic visualization of 3D CT-IV images in situ superimposed on the surgical area with the naked eye. Dental education using VR in the field of dental anatomy was presented by Libermann and Erdelt [30]. It was found that the proposed solutions helped over 90% of the students to better understand the presented subject. In turn, Morales-Vadillo et al. [31] described the use of the online virtual world with a social environment to further explore the topic of dental clinical situations. Their study suggests that this helps students to understand the anatomical interaction better than in the traditional model of dental education.

Our results show that AR- and holography-based technology can be successfully used in student education, especially in the area of dental root anatomy. It allows consultations with experts from different fields and sharing 3D images in real time. Students have the opportunity to learn dental morphology and pathology not only by reading or watching a video, but also by immersing themselves in the enhanced transparency. Although our application refers to a part of dental anatomy, the high prevalence of endodontic problems in many countries and the difficulty of learning root canal morphology suggest that the use of our invention may be justified. Moreover, dental morphology is only one possible application. The future development of holography and AR-based training system may help to create an operating room with unlimited perspective that can be used in the training system for students, especially in the field of dental implant surgery. The application of immersive technologies can have a positive impact on the results of dynamic navigation.

Despite the potentially promising and broad application of holographic technology in medicine and dentistry, it is also important to mention its drawbacks such as its relatively high price and the challenges of ensuring proper hygiene when using the HoloLens2 goggles. Regarding the high price, it can be expected to drop as it is commonly seen with the spread of the technology or device. Disinfecting with liquids every time and using disposable gloves could provide an adequate level of hygiene. Other potential disadvantages include development of mild eyestrain, headaches, or motion sickness in some students [32]. Of course, there is no substitute for face-to-face contact with the teacher, but especially in times of a pandemic, this technology offers reasonable alternative.

It is also important to note that this study only tested to a limited extent the effectiveness of the developed application as an adjunct in the dental education process.

## Data Availability

Not applicable.
